# The impact of Hounsfield unit-related variables on percutaneous nephrolithotomy outcomes

**DOI:** 10.1038/s41598-022-23383-7

**Published:** 2022-11-02

**Authors:** Hyong Woo Moon, Mustafa Taeyb, Yong Hyun Park, Woong Jin Bae, U.-Syn Ha, Sung-Hoo Hong, Ji Youl Lee, Sae Woong Kim, Hyuk Jin Cho

**Affiliations:** 1 Department of Urology, Seoul St. Mary’s Hospital, College of Medicine, The Catholic University of Korea, 222, Banpo-Daero, Seocho-Gu, Seoul, 06591 Republic of Korea; 2 Department of Urology, King Faisal Hospital, Mecca, Saudi Arabia

**Keywords:** Nephrology, Urology

## Abstract

We aimed to identify the association between Hounsfield Unit(HU)-related variables and percutaneous nephrolithotomy (PCNL) outcomes. We enrolled patients with single renal stones (1–3 cm) who underwent single-tract PCNL between January 2014 and October 2019. Demographics and stone characteristics were retrospectively reviewed. Preoperative computerized tomography (CT) and follow-up CT within at least 3 months after PCNL were included in this analysis. Stone-free status was defined as residual stone measuring ≤ 2 mm within 3 months postoperatively. HU and cross-sectional area (CSA) were measured using the free-draw technique. We analyzed HU-related variables using logistic regression model for outcomes. Altogether, 188 out of 683 patients met the inclusion criteria. The stone-free rate (SFR) was 79.2%. There were no significant differences in age, sex, BMI, ASA class, laterality, pre-op shockwave lithotripsy, stone size, stone burden, skin-to-stone distance, and HU between the stone-free and remnant groups. CSA and HU/CSA in the stone-free and remnant groups were 94.5 ± 46.1 and 128.3 ± 98.5 (p = 0.043) and 10.1 ± 5.6 and 7.3 ± 3.4 (p = 0.001), respectively. Multivariate logistic regression analysis revealed that pelvis, ureteropelvic junction stones, and HU/CSA were independent predictors of SFR. HU did not affect PCNL outcomes. We believe that HU/CSA could be used for determining stone treatment plans and predicting outcomes.

## Introduction

Noncontrast computed tomography (NCCT) is widely accepted for diagnosing urolithiasis. It has higher sensitivity (> 94%) and specificity (> 95%) than plain radiography ultrasonography and intravenous pyelography^1^. The information obtained from NCCT to determine treatment modality includes stone size, multiplicity, location, anatomic anomaly, skin-to-stone distance, stone density (Hounsfield unit [HU]), and fragility (stone heterogeneity index [SHI] and variation coefficient [VCSD])^2–5^. The size of the stone is important in predicting the possibility of spontaneous passage. HU, SHI, and VCSD are related to stone composition and fragility, and they determine the success of shock wave lithotripsy (SWL). However, there are few studies in the literature regarding the predictive role of HU in the outcomes of percutaneous nephrolithotomy (PCNL)^6–9^.

The stone scoring systems used to predict the outcome of PCNL are the STONE, CROES nephrolithometric nomogram, and Guy’s stone score^10–12^. These scoring systems were suggested based on the stone number, HU, burden, location, anatomical abnormality, and the surgeon’s experience. Of these scoring systems, only the STONE scoring system includes HU; however, in this scoring system, the clinical evidence for setting HU (950) as the cut-off value is insufficient.

This study aimed to identify the association between the HU-related variables and outcome of PCNL. We introduced the concept of stone density based on HU and cross-sectional area (CSA) as a possible predictor of PCNL outcome.

## Materials and methods

### Ethics approval

This study was approved by the Institutional Review Board of The Catholic University of Korea (approval number KC18RESI0836). All procedures performed in study involving human participants were in accordance with the ethical standards of the institutional and/or national research committee and with the 1964 Helsinki Declaration and its later amendments or comparable ethical standards. All information used for statistical analysis was anonymized, and the requirement for obtaining informed consent was waived by the Institutional Review Board of The Catholic University of Korea.

### Patient selection and study design

**Figure 1:**
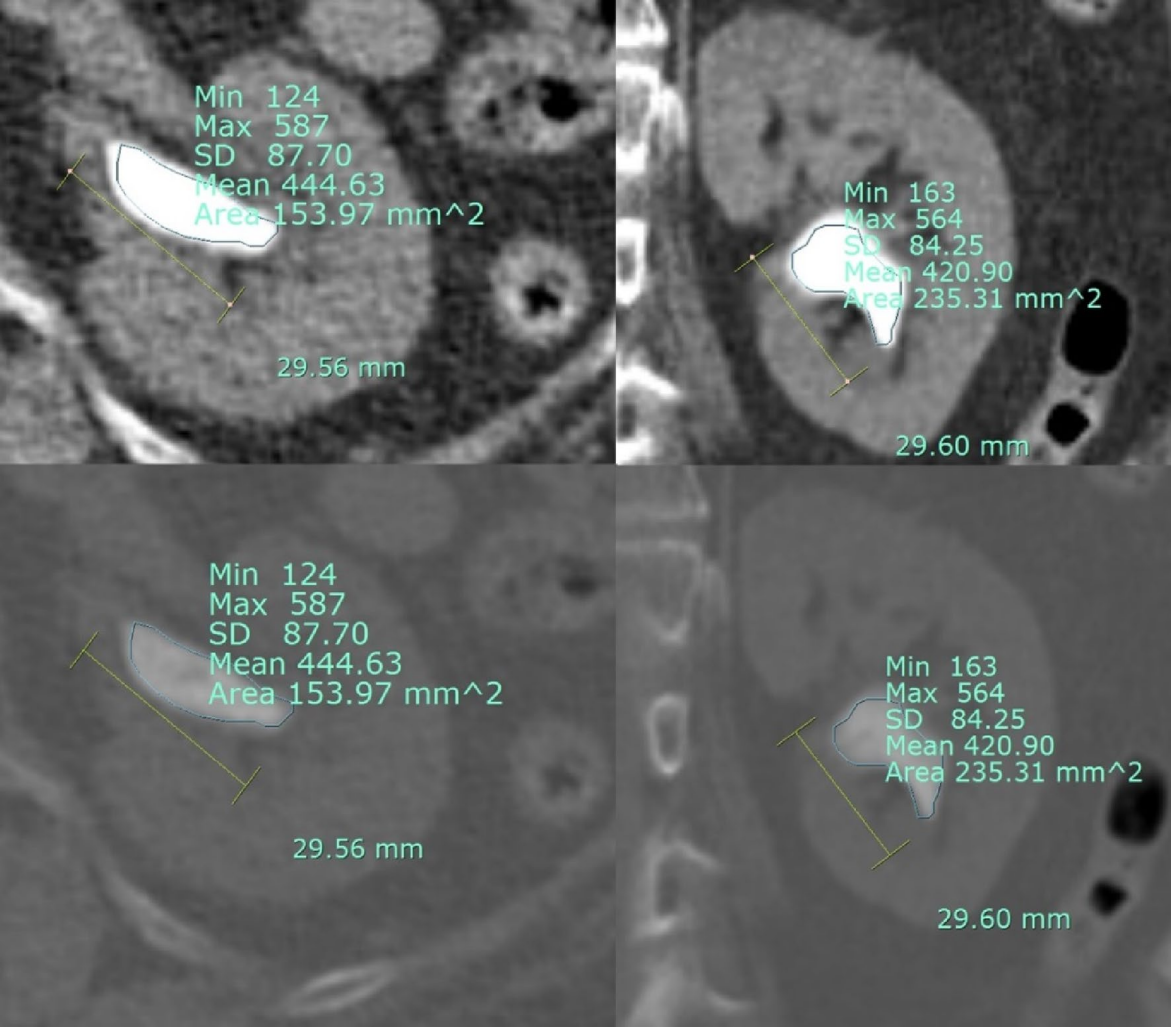
Free draw measurement technique of renal stone. Axial vs. coronal view, and soft tissue setting vs. bone setting view. In soft tissue and bone setting views, the stone has almost similar Hounsfield unit and stone heterogeneity index values. In the axial and coronal views, the value of the maximal stone diameter is equal to 29 mm; however, the cross-sectional areas are 153 and 235 mm^2^, respectively.

We retrospectively reviewed data of 683 consecutive patients who underwent PCNL at a single tertiary institution between January 2014 and October 2019. Overall, 188 patients with single-tract PCNL and a maximal stone diameter of 10–30 mm were included and evaluated with pre- and postoperative CT scans. Our study analyzed the impact of HU-related variables on PNL, Therefore, the stone size, which is generally known to have a significant impact on success rates, was limited to single stones with 10–30-mm size. The 10–20-mm sized stones in the lower calyx were included according to the stone management guidelines^13^. We retrospectively evaluated the patients’ characteristics, stone-related variables measured by NCCT, and operation parameters (operative (OP) time, stone-free rate, hemoglobin, hematocrit, creatinine, hospital stay, and complications). The stone-related variables were stone diameter, stone location [ureteropelvic junction (UPJ), renal pelvis, lower/mid/upper calyx], stone maximal CSA, skin-to-stone distance, HU value using the free-draw method at the maximal CSA, and the SHI (Standard deviation of HU). Among the axial and coronal views, the larger CSA was set as the reference value at the bone setting view. The free-draw method is done easily using the PACS program by utilizing digital free-hand calipers (Fig. 1). Moreover, it was measured by directly laying it out using a free hand along the inner layer of the stone shape in maximal CSA. Two urologists, including a urologic resident, evaluated the stone status and performed stone measurement and calculation.

We defined stone-free status (SFS) as the absence of residual fragments or presence of a clinically insignificant remnant stone (≤ 2 mm). OP time was defined as the time from percutaneous access puncture to the final dressing at the incision site.

Post-PCNL outcome data were retrospectively collected from electronic medical records. Pre- and postoperative radiologic evaluation of the kidneys, ureter, and bladder (KUB) and NCCT examinations were performed in all patients. All patients who underwent PCNL were evaluated by plain KUB radiography and NCCT within 3 months postoperatively. All cases were analyzed at 1 week, 4–8 weeks, and 3–6 months postoperatively. Patients with remnant stone fragments were followed up every 6 months after routine follow-up.

All procedures were performed by experienced endourologist who performed more than 150 cases of PCNL in a year. The exclusion criteria for this study were simultaneous bilateral PCNL and multiple approaches requiring more than two nephroscopic tracts. Perioperative variables and baseline demographics were compared between patients with SFS and remnant status. The preoperative data included body mass index, American Society of Anesthesiology classification, and preoperative SWL (Table 1). The operative data and complication data were also reviewed. Complications were recorded according to the modified Clavien system for reporting complications^14^. The primary endpoint was to calculate the correlations between HU-related variables and PCNL outcomes.

**Table 1:** Demographic data in the stone-free and remnant groups.

	Stone-free (N = 149)	Remnant (N = 39)	*p*
Male	103 (69.1%)	23 (59.0%)	0.313
Age (years)	55.0 ± 12.7	55.3 ± 14.4	0.892
BMI	24.8 ± 3.5	24.0 ± 3.4	0.241
**ASA class**			0.925
2	92 (61.7%)	25 (64.1%)	
3	10 (6.7%)	2 (5.1%)	
Laterality (Rt)	87 (58.4%)	17 (43.6%)	0.140
Hydronephrosis	46 (56.1%)	14 (46.7%)	0.501
Pre OP SWL	30 (20.1%)	6 (15.4%)	0.658
Pre OP pyuria	65 (43.6%)	20 (51.3%)	0.500
Upper calyx stone	10 (6.7%)	5 (12.8%)	0.357
Mid-calyx stone	8 (5.4%)	2 (5.1%)	1.000
Lower calyx stone	38 (25.5%)	19 (48.7%)	0.009
Pelvic or UPJ stone	93 (62.4%)	13 (33.3%)	0.002
SSD mean (mm)	87.1 ± 15.6	85.6 ± 15.1	0.608
Stone size (mm)	16.8 ± 4.6	17.4 ± 5.2	0.515
Stone cross-sectional area (mm^2^)	94.5 ± 46.1	128.3 ± 98.5	0.043
Stone burden (mm^2^)	163.5 ± 113.7	177.2 ± 110.8	0.503
Radio opacity	120 (80.5%)	30 (76.9%)	0.782
Hounsfield unit (HU)	769.2 ± 304.9	750.2 ± 349.6	0.737
Stone heterogeneity index (SD)	213.7 ± 110.7	203.0 ± 126.7	0.603
HU density (HU/mm)	48.0 ± 20.1	43.5 ± 19.2	0.216
HU density (HU/mm^2^)	10.1 ± 5.6	7.3 ± 3.4	0.001
Variation coefficient (%)	28.3 ± 12.9	27.6 ± 12.3	0.765

*SHI* stone heterogeneity index; standard deviation of Hounsfield unit.

Variation coefficient: Hounsfield unit/standard deviation of Hounsfield Unit × 100 (%).

### Surgical technique

Urinalyses and cultures were obtained preoperatively for all patients, and prophylactic antibiotics were administered according to the results.

The procedures were performed with the patients in prone position after undergoing cystoscopic ureteral catheter insertion, which was carried out under general anesthesia. The Foley catheter is inserted after ureteral catheterization. It is then placed in a sterile bag with a ureteral catheter to prevent dislocation and contamination before prone position. Foley catheter is removed on postoperative day 1. Percutaneous access was achieved using fluoroscopic guided puncture, with balloon dilatation up to 24 Fr. A 20-Fr nephroscope was used for the procedure. The stone was fragmented using pneumatic and ultrasonic devices (Swiss Lithoclast, EMS Electro Medical System, Switzerland). Once PCNL was completed, the collecting system was carefully inspected, and a nephrostogram was performed to exclude remnant stones. The procedure was terminated without nephrostomy tube insertion, unless there were any intraoperative events or suspected residual stones. D–J insertion was not performed routinely unless there was an edematous UPJ, suspected remnant stones, or significant bleeding during the procedure^15^.

### Statistical analyses

Continuous variables are reported as mean ± SD or range and were compared using independent sample Student’s *t*-test or Mann–Whitney U test as appropriate. Categorical variables were analyzed for statistical significance using Chi-square or the linear-by-linear association test. We calculated using the G*Power 3.1 Window program, required number of samples for logistic regression was at least 158 (α error 0.05, power 1 − β = 0.80, Odds Ratio (OR) = 0.6, and Pr(Y = 1 | X  = 1)H0 = 0.3), making the number of subjects in this study sufficient to obtain power. We used a logistic regression model to perform univariate and multivariate assessments to determine the predictors of SFS and OP time. *p*-values < 0.05 were considered statistically significant. Inter observer reliability was assessed with the intraclass correlation coefficient (ICC)^16^. Statistical analysis was performed using R software (version 3.3.2, R Foundation for Statistical Computing, Vienna, Austria; http://r-project.org). G power 3.1 for window.

## Results

### Patient demographics, stone characteristics, and perioperative data

From January 2014 to October 2019, 683 patients underwent PCNL. Subsequently, 495 were excluded due to stone number, size, and tract number. Finally, 188 patients met the inclusion criteria. After PCNL, the SFS rate was 79.2% (149/188) and the remnant rate was 20.8% (39/188). Demographics, operative factors, and stone characteristics were compared between the stone-free and remnant groups (Tables 1, 2); significant differences were found in lower calyx stone (25.5% vs. 48.7%, p = 0.009), pelvis and UPJ stone (62.4% vs. 33.3%, p = 0.002), stone CSA (94.5 ± 46.1 vs. 128.3 ± 98.5, p = 0.043), HU/CSA (10.1 ± 5.6 vs. 7.3 ± 3.4, p = 0.001), and OP time (56.4 ± 22.6 vs. 6.55 ± 22.9, p = 0.027). The modified Clavien–Dindo classification was used for complication categorization (Table 3). In total, 31 complications occurred in 25 (13.3%) patients.

**Table 2:** Operative factors in the stone-free and remnant groups.

	Stone-free (N = 149)	Remnant (N = 39)	*p*
OP time (mins)	56.4 ± 22.6	65.5 ± 22.9	0.027
Access time (mins)	7.7 ± 4.4	7.6 ± 5.0	0.939
Total nephroscopy time (mins)	18.3 ± 10.4	25.9 ± 17.1	0.072
Subcostal approach	111 (74.5%)	28 (71.8%)	0.891
Antegrade D–J catheter	70 (46.98%)	15 (38.46%)	0.441
LOS (days)	3.4 ± 3.7	2.8 ± 1.2	0.096
Complication (n)	18 (12.2%)	7 (17.9%)	0.496
Transfusion (n)	4 (2.68%)	0	0.586
Change in Hct (%)	− 6.4 [− 7.7; − 3.6]	− 6.3 [− 8.9; − 4.9]	0.305
Change in Hb (g/dL)	− 2.0 [− 2.8; − 1.2]	− 2.1 [− 3.0; 1.7]	0.383
Change in Cr (mg/dL)	− 0.10 [− 0.21; 0.03]	− 0.09 [− 0.15; 0.11]	0.209
Change in eGFR (1 month) mL/min/1.73 m^2^	− 10.5 [− 21.0; 1.0]	− 6.0 [− 15.5; 2.5]	0.286

*SHI* stone heterogeneity index; standard deviation of Hounsfield unit.

Variation coefficient: Hounsfield unit/standard deviation of Hounsfield Unit × 100 (%).

**Table 3:** Perioperative complications according to the modified Clavien classification system.

Complication grading	No. of complications (%)
**Grade I**	**16 (51.6%)**
Postoperative pain managed by opioid with adjunct analgesic regimen	6
Postoperative fever (> 38.0 °C) managed by observation without antibiotics	6
Bladder retention without blood clot that requires bladder catheterization	1
Urine leakage managed by watchful waiting	2
Bleeding that requires a single episode of nephrostomy clamping	1
**Grade II**	**9 (29.0%)**
Bleeding requiring blood transfusion	4
Symptomatic UTI managed using antibiotics	3
Minor atelectasis requiring medical management	2
**Grade IIIa**	**4 (12.9%)**
Febrile UTI or suspected sepsis without organ failure requiring supportive therapy and enhanced monitoring	1
Colon perforation managed conservatively using controlled colocutaneous fistula	1
Hemothorax managed by intercostal draining under local anaesthesia	1
Ureteric clot obstruction managed by ureteric stenting without general anaesthesia	1
**Grade IIIb**	**1 (3.2%)**
Bleeding managed by angioembolization	1
**Grade IVb**	**1 (3.2%)**
Urosepsis with multiple organ failure requiring ICU management	1
**Grade V**	**0**
Any complication leading to death	0
Total complications	31 complications in 25 pts (13.3%)

*UTI* urinary tract infection, *ICU* intensive care unit.

### Multivariate logistic regression of SFR

In the univariate analysis, the following factors were significantly related to remnant status: lower calyx stone (p = 0.006, Odds ratio = 2.77), pelvis or UPJ stone (p = 0.002, Odds ratio = 0.30), stone CSA (p = 0.013, Odds ratio = 2.77), and HU/CSA (p = 0.00, Odds ratio = 0.87). In a final model of the multivariate logistic regression analysis, pelvis and UPJ stone (p = 0.003, Odds ratio = 0.65) and HU/CSA (p = 0.027, Odds ratio = 0.90) were independent significant predictors of SFS in overall patients (Table 4). Lower calyx stone, SSD, HU, SHI, Radio-opacity did not affect the SFS. The stone status prediction logistic regression model was shown to be significant (× 2 = 22.497, p = 0.001), and the Nagelkerke coefficient of determination (R^2^) of the model showed a 48.7% explanatory power.

**Table 4:** Univariate and multivariate logistic regression analyses of stone-free status after single-tract and single-stone PCNL.

Stone-free	Univariate analysis			Multivariate analysis		
	OR	95% CI	*p*	OR	95% CI	*p*
Lower calyx stone	2.77	1.34–5.75	0.006			
Pelvis + UPJ stone	0.30	0.14–0.63	0.002	0.65	0.14–0.66	0.003
Skin-to-stone distance	0.99	0.97–1.02	0.606			
Hounsfield unit	1.00	1.00–1.00	0.513			
Stone cross-sectional area (mm^2^)	1.01	1.00–1.02	0.013	1.01	1.00–1.01	0.091
Stone heterogeneity index (SD)	1.00	1.00–1.00	0.601			
Hounsfield unit density (HU/CSA mm^2^)	0.87	0.79–0.96	0.005	0.90	0.81–0.99	0.027
Radio-opacity	0.81	0.34–1.88	0.617			

## Discussion

In this study, we focused on the association between HU-related variables and PCNL outcome measured by CT using the free-draw stone measurement method. The main study findings were as follows: factors that affect single-stone PCNL SFR are stone location and HU/CSA; and the factor that affects OP time is the CSA.

For the association between HU and the outcome of PCNL, Gucuk et al.^6,9^ reported that the cut-off value of HU should be 677.5, and the success rate decreases if the HU value is low. Meanwhile, Alper et al.^7^ suggested that there is no correlation between HU, success rates, and OP time, and that fluoroscopy time is prolonged when the HU value is high. The only large-scale study was conducted by CROES Group in 2013 using a multicenter design; the authors reported that the success rate decreased for very low- and high-density stones, and longer OP times were required^8^. As such, there is no consensus regarding the effects of HU on PCNL outcomes. The major reason for inconsistency is the lack of inter-observer reliability and reproducibility because of different methods for measuring HU.

In the present study, the location that affected stone-free rate was the renal pelvis; these stones are graded I in the Guy's stone scoring system. Using HU/CSA as the predictor of PCNL outcome, we demonstrated a tendency toward a high stone-free rate for stones that have high HU/CSA values. This can be explained by the fact that SFR would be high as HU increases in the same CSA and that SFR would be high as CSA decreases in the same HU.

In the CROES study^8^, PCNL success probability was low for very low- and high-density stones. First, that study did not employ a consistent measurement methodology across a large number of centers. Second, there was a heterogeneity in terms of stone assessment due to the differences in CT slice thickness and energy protocols. Third, HU tends to increase in proportion to the size of the stone^17^ and the proportion of radio-opacity increases as HU increases. Considering all of these points, it would be insufficient to believe that the effect of HU itself on OP time is significant. In our study, there were no significant differences in terms of analysis of OP time based on HU, radio-opacity, and stone heterogeneity index. Furthermore, there was no significant correlation between OP time and HU as a continuous variable.

Considering stone size measurement, Patel et al. raised issues regarding limits because of high inter-observer variability from the existing manual measurement and the proposed effectiveness of stone measurement methods using commercialized computer programs^18,19^. Nevertheless, there is insufficient clinical or cost-related evidence to start using it in clinical practice. On the other hand, regarding the free-draw measurement we used, Tanaka et al.^20^ suggested the efficiency of the measurement using stone CSA and using it to predict the SWL outcome.

In terms of standardization of HU measurement, Narayan et al.^21^ compared region-of-interest measurement techniques. There appears to be no consensus regarding which measurement technique is effective. Motley et al.^22^ and Nakada et al.^23^ reported that HU and stone diameter ratio were important factors for predicting calcium stone and uric acid stone outcomes. To the best of our knowledge, the present study is the first to report the ratio of HU and CSA and its effect on PCNL outcomes.

Understanding that there is no standard measurement method, our free-draw technique can be used as a single measurement in almost all PACS programs. Moreover, we limited our study population to a single stone that is larger than the size of CT thickness and minimized the partial volume effect. The homogeneity of this dataset represents an advantage of the present study.

Despite these advantages, our study has several limitations. First, it has a retrospective design with a small number of patients. Although, the stone range (1.0–3.0 cm) makes the group heterogeneous, it can be considered that bias was minimized because it was targeted for single-stone and single-tract PCNL patients. Recently, along with a growing trend of offering patients with ‘decided choice’ of surgery, it is important to note that there is a possibility of offering flexible ureteroscopy in patients with stones with < 2 cm in size or even a ‘combo approach’ with SWL and flexible ureteroscopy^24^. Second, there is a possibility that the CSA/HU ratio as the predictor could be a confounding variable. Because HUs tend to increase with increasing stone size^25^, CSA alone may be the factor for determining SFR. Moreover, it possible that a measurement bias exists between the physicians using the free-draw stone measurement method. However, in measuring the Hounsfield unit, it is known that inter/intra observer variability shows a high probability of agreement regardless of the measurement method^26^, and our data also showed agreement in intraclass correlation coefficient analysis. Nevertheless, maximal stone diameter, CSA, and HU were not significant predictive factors for SFR, and we minimized the possibility of confounding variables using multivariate logistic regression. There are many variables that affect PCNL outcome, our study group contains only single stone and single tract PCNL patients. Finally, we did not include the analysis of stone composition. For these reasons, a prospective study on larger numbers is required, with the analysis of stone composition included.

In conclusion, there was no effect of HU itself on PCNL outcome. However, a HU-related variable, HU/mm^2^, was predictive of SFR. We believe that HU/CSA could be used to design stone treatment plans and to predict PCNL outcomes. With future research, HU/CSA could be used as an effective variable in determining ureteroscopic surgery and SWL outcome.

## Data Availability

The datasets generated during and/or analyzed during the current study are available from the corresponding author on reasonable request.
